# Factors Influencing Telehealth Service Use and Health Outcomes in Patients Undergoing Continuous Ambulatory Peritoneal Dialysis: Cross-Sectional Study

**DOI:** 10.2196/48623

**Published:** 2023-12-05

**Authors:** Nattaya Praha, Aurawamon Sriyuktasuth, Wimolrat Puwarawuttipanit, Piyatida Chuengsaman, Worapan Kusakunniran

**Affiliations:** 1 Faculty of Nursing Mahidol University Bangkok Thailand; 2 Banphaeo Dialysis Group Banphaeo Hospital Bangkok Thailand; 3 Faculty of Information and Communication Technology Mahidol University Nakhon Pathom Thailand

**Keywords:** acceptance, cross-sectional study, health system and access, mHealth, mobile health, peritoneal dialysis, telehealth

## Abstract

**Background:**

Several studies have demonstrated the efficacy and user acceptance of telehealth in managing patients with chronic conditions, including continuous ambulatory peritoneal dialysis (CAPD). However, the rates of telehealth service use in various patient groups have been low and have declined over time, which may affect important health outcomes. Telehealth service use in patients undergoing CAPD has been recognized as a key challenge that needs to be examined further.

**Objective:**

This study aimed to explore the rates of telehealth service use over 4 months, identify factors influencing its use, and examine the relationship between telehealth service use and health outcomes in Thai people undergoing CAPD.

**Methods:**

This cross-sectional study, which was a part of a pragmatic randomized controlled trial study, was conducted at a dialysis center in Bangkok, Thailand. The study included patients who were undergoing CAPD. These patients were randomly enrolled in the intervention group to receive telehealth service and additional standard care for 4 months. Data were collected using self-reported questionnaires, including a demographic form, Functional, Communicative, and Critical Health Literacy Scale, Perceived Usefulness Questionnaire, Brief Illness Perception Questionnaire, Patient-Doctor Relationship Questionnaire, and Kidney Disease Quality of Life 36 Questionnaire. Additionally, Google Analytics was used to obtain data on the actual use of the telehealth service. These data were analyzed using descriptive statistics, repeated-measures ANOVA, and regression analyses.

**Results:**

A total of 159 patients were included in this study. The mean rate of telehealth service use throughout the period of 4 months was 62.06 (SD 49.71) times. The rate of telehealth service use was the highest in the first month (mean 23.48, SD 16.28 times) and the lowest in the third month (mean 11.09, SD 11.48 times). Independent variables explained 27.6% of the sample variances in telehealth service use. Older age (β=.221; *P*=.002), higher perceived usefulness (β=.414; *P*<.001), unemployment (β=−.155; *P*=.03), and positive illness perception (β=−.205; *P*=.004) were associated with a significantly higher rate of telehealth service use. Regarding the relationship between telehealth service use and health outcomes, higher rates of telehealth service use were linked to better quality of life (β=.241; *P*=.002) and lower peritonitis (odds ratio 0.980, 95% CI 0.962-0.997; *P*=.03).

**Conclusions:**

This study provides valuable insights into factors impacting telehealth service use, which in turn affect health outcomes in patients undergoing CAPD.

## Introduction

Continuous ambulatory peritoneal dialysis (CAPD) is a home-based therapy performed autonomously by trained patients or with the assistance of caregivers. Although CAPD offers several benefits for patients and the health care system [1,2], the patients inevitably face numerous complications, particularly peritonitis [3,4], which has been identified as a core outcome of peritoneal dialysis (PD) care [5,6]. This complication is a key reason for switching the mode of dialysis [7,8] and is associated with substantial morbidity [9], which consequently diminishes the quality of life [10,11]. However, the current PD care service does not allow health care providers to closely monitor PD exchanges and patients’ conditions while administering the treatment at home [12]. Therefore, integrating telehealth into health services could enhance patient education and facilitate access to care [13]. Telehealth is an effective tool to monitor patients and support self-management and self-monitoring [13-15], thereby enhancing the feeling of safety at home [16]. Additionally, telehealth has the potential to support health care delivery and fulfill health care needs in patients with chronic diseases at all levels of care [17], particularly in those receiving home-based treatments such as CAPD [15,16].

Although telehealth service use among patients undergoing CAPD and having other chronic conditions showed high acceptance [18-21], the rates of telehealth service use among patients as end users were low and fragmented [22-28]. This finding is inconsistent with the rapid advances in health and communication technology. Patients’ use is a key factor in telehealth success and is one of the important challenges [29]. However, factors that drive the use of home-based telehealth services from a consumer perspective have not been adequately investigated. This gap highlights the need to explore the key factors that either motivate or hinder the use of telehealth services for patients with CAPD. Increased use of telehealth services can result in a shift in the health care paradigm from conventional to active and progressive health management, ultimately improving CAPD care quality.

Health care service use, particularly telehealth service, might be affected by various factors. The Andersen behavioral model [30] was developed to understand the determinants of health care service use. This model includes multidimensional factors associated with health care services and their use. These factors are categorized as predisposing characteristics, enabling resources, needs, and environmental factors. The model also proposed that these factors influence health care service use and impact health outcomes, which are the end points of interest [30].

Predisposing characteristics are defined as personal qualities and beliefs about or attitudes toward health care services [30]. With regard to the prevailing knowledge about the predisposing factors influencing the use of telehealth services, findings from previous studies vary. Likewise, predisposing characteristics, such as age, were not related to telehealth service use in Thai [31] and American populations [27]. Conversely, several studies observed an association between a higher rate of telehealth service use and younger age [32-36], whereas other studies revealed an association with older age [28,37,38]. Furthermore, health literacy, a predisposing factor, was important in the delivery of effective health care service [39,40]. Previous investigations reported that sufficient health literacy was associated with telehealth service use [41-45]. On the contrary, a study by Manganello et al [46] showed that individuals with low self-reported health literacy were more likely to use telehealth service through mobile health app. Other studies, however, failed to identify a link between health literacy and the use of telehealth service and other health care services in promoting health behaviors [47,48]. Furthermore, perception of telehealth services, particularly perceived usefulness, was found to be a strong influencing factor in telehealth service use, as confirmed by previous studies [18,19,49-52], systematic reviews, and meta-analyses [20,53,54]. Nonetheless, most previous investigations focused on the acceptance or intention to use or self-reports in using those telehealth services provided for other populations with different health conditions and telehealth platforms.

Enabling resources refer to states that allow an individual to act on a value or meet a need related to the use of health services such as financial resources, social support, and other personal resources [30]. Employment status is considered an enabling resource in terms of the time available to use telehealth services. However, there have been paradoxical findings in previous studies about the association between telehealth service use and employment status [32,34,55,56], which cannot be used to draw a conclusion.

Need captures illness-related factors, such as the type and nature of the illness, or health conditions that necessitate health service use [30]. The influence of need factors on telehealth service use was found to be controversial in previous studies. For instance, higher use of telehealth services was associated with more comorbidities [32,34] and higher health demands [56], whereas other studies observed higher use in patients with good health status and good physical activity [27,57,58]. The relationship between patients’ disease and the use of telehealth services has been rather inconclusive.

Environmental factors, which are defined as the circumstances and context related to health organizations and health care providers, also have an impact on telehealth service use. Relationships between patients and health care providers are associated with patients’ satisfaction and hence lead to effective and successful delivery of health care services [59-61]. Findings from previous studies revealed that a better relationship between health care providers and patients was related to the use of telehealth services [50,62]. However, these studies were conducted in different populations, health care contexts, telehealth services, and cultural contexts. Therefore, there is a need to explore the influence of the relationship between patients and health care providers on telehealth service use in patients with CAPD who require strong support from health care providers while performing dialysis at home.

As mentioned above, existing reports have used various telehealth services with varying platforms in different populations. Hence, the factors associated with those telehealth service uses cannot be concluded. Most studies were conducted under other chronic conditions and in the general population living in developed countries. Higher-income countries show more telehealth activities than lower-income countries [32,55,57,63], which may affect the study results owing to various differences, such as society, culture, economy, infrastructure, health care system, and technological advancement. There have been limited studies or reports on people with CAPD. Moreover, few studies have simultaneously examined actual telehealth service use and its potential influencing factors. Additionally, few researchers have integrated theoretical frameworks to guide their studies [29]. To the best of our knowledge, no study has focused on telehealth service use among patients with CAPD. Thus, additional studies are needed to explore the rate of telehealth service use and its influencing factors in patients with CAPD.

To address these gaps, we aimed to examine the rate of telehealth service use and identify potential factors associated with it. We further explored the relationship between these rates and important health outcomes in patients with CAPD. Informed by the Andersen behavioral model, modifiable factors, including predisposing characteristics (age, health literacy, and perceived usefulness), enabling resources (employment status), need factors (perception of illness), and environment factors (patient-provider relationship), were selected to identify the factors associated with telehealth service use. Additionally, the relationships between the use of telehealth services and health outcomes of patients (quality of life and peritonitis) were explored. The findings of this study may be helpful in enhancing telehealth service use. Furthermore, identifying the relationship between the rates of telehealth service use and health outcomes may aid in determining the optimal level of adherence use of the health service in this population.

## Methods

### Study Population

This cross-sectional study was conducted as a part of the pragmatic randomized controlled trial (RCT) study at the Banphaeo Dialysis Center, Bangkok, Thailand. Eligibility criteria included treatment with CAPD, age ≥18 years, ability to communicate in Thai, access to a smartphone or tablet capable of running the PD Easy app, and using the telehealth service for 4 months. Vulnerable individuals with serious chronic illnesses, such as malignancy, severe heart failure, major mental illnesses, and cognitive disorders, were excluded.

### Sampling and Sample Size

The RCT aimed to examine the effects of telehealth service on health outcomes and enrolled 338 patients who were undergoing CAPD for at least 3 months to ensure adequate experience and time to settle into this treatment. Before recruitment to the RCT study, a statistician created a table of random numbers by using computer-generated sequences. The sample was conveniently selected with the eligible criteria. Interested patients were screened for eligibility by PD nurses. Following informed consent, the participants completed a demographic form and baseline questionnaires. Subsequently, participants were randomly allocated to either the intervention or the control group using sealed envelopes containing nonduplicated numbers (1-338). All 338 participants were randomly assigned into 2 groups for 12 months: the intervention group (telehealth service plus standard care; n=169) and the control group (standard care only; n=169). RCT outcomes were evaluated at baseline, 4 months, and 12 months. As part of the RCT study, all eligible participants who were randomly assigned to the intervention group for receiving telehealth service plus standard care for 4 months were conveniently included in this study. Based on the eligibility criteria, a total of 159 participants were recruited in this study. Although the formal sample size for this study was not calculated, a minimum sample size of 158 was required based on the power analysis for multiple regression with a power of 0.80, a significance level (α) of .05 (*P* value), and 6 independent variables, as well as an effect size of 0.09 from a previous study [64]. Therefore, the sample size used in this study can be considered adequate.

### Telehealth Service and Its Features

**Figure 1:**
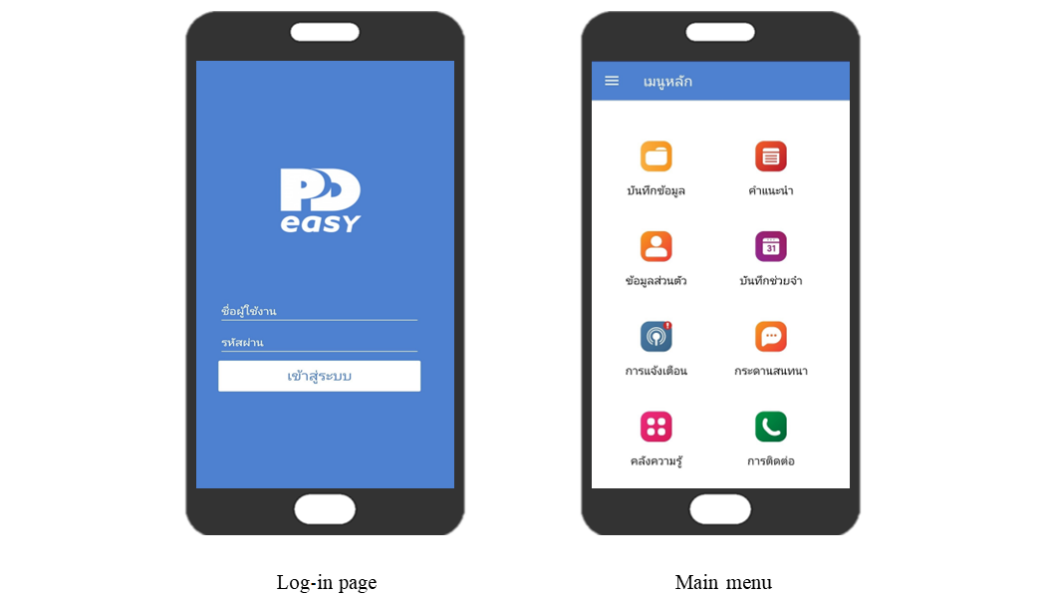
Sample screenshots of the telehealth service (PD Easy mobile app).

**Figure 2:**
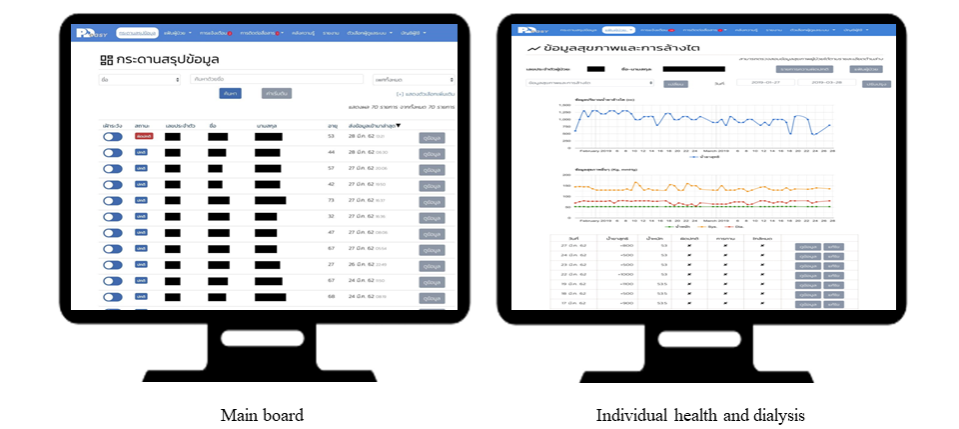
Sample screenshots of the telehealth service (PD Easy web application).

A total of 159 participants enrolled in this study received telehealth service operated through a mobile (patient side) and web application (dialysis center side) called PD Easy for 4 months plus standard care. The mobile app comprised 8 core features, including (1) daily health and dialysis records, (2) information, (3) health advisory, (4) reminders, (5) health alerts, (6) social forum, (7) news and knowledge management, and (8) contacts (Figure 1). More information about the telehealth service is available elsewhere [21]. The telehealth service was operated by a multidisciplinary team comprising nephrologists, PD clinic nurses, public health technical officers, and a dietitian to provide self-management support, monitor home dialysis, and enhance patient health professional communication for patients undergoing CAPD (Figure 2). A total of 2 health care providers acted as PD web application administrators. They reviewed and monitored information submitted by the participants to the PD telehealth system and coordinated with other health care providers to manage or solve any problems during the course of home-based treatment.

### Study Variables

The independent variables included age, health literacy, perceived usefulness, employment status, perception of illness, and patient-provider relationship.

Telehealth service use and health outcomes (quality of life and peritonitis rate) were the dependent variables.

### Measurements and Data Collection

Demographic and health data were retrieved from the demographic form and medical records, respectively. Telehealth service use was measured by recording the number of log-ins to the mobile app over 4 months using Google Analytics. This analytical tool was used to collect use data for the telehealth service operated through web and mobile applications. Therefore, the actual telehealth service use by the participants was obtained for data analysis. Health literacy was measured using the Functional, Communicative, and Critical Health Literacy Scale [65] and perceived usefulness was measured using the Perceived Usefulness Questionnaire [66]. The perception of illness was assessed using the Brief Illness Perception Questionnaire [67] and the patient-provider relationship was determined using the Patient-Doctor Relationship Questionnaire [68]. Health outcomes included the quality of life (measured using the Kidney Disease Quality of Life 36) [69] and peritonitis (extracted from the participant’s medical records).

All questionnaires were well-developed, used in previous studies, and validated by experts. Therefore, validity testing was not performed in this study. In addition, for all instruments, a Thai version was available for use by the Thai population, except for the Perceived Usefulness Questionnaire. To use this scale with Thai patients with CAPD, the researchers translated it into the Thai language using the back translation method. Permissions to use all scales were obtained from the original tool developers and translators.

The instrument’s reliability was assessed with a separate sample of 30 participants who were similar to the sample in the study. All instruments were not changed after the pilot test for reliability. The Cronbach α of Functional, Communicative, and Critical Health Literacy Scale was .80, Perceived Usefulness Questionnaire was .90, Brief Illness Perception Questionnaire was .87, Patient-Doctor Relationship Questionnaire was .95, and Kidney Disease Quality of Life 36 was .92. Data were collected 4 months after enrollment in the RCT study using self-reported questionnaires.

### Ethical Considerations

This study was approved by the institutional review board for human participants research at the Faculty of Nursing, Mahidol University (IRB-NS 2020/547.1802). Participants were informed about the study objectives, protocols, benefits, risks, privacy, and confidentiality. All participants provided informed consent. To ensure the confidentiality of the participants, they received a unique identification code that was used for all questionnaires. All data were securely maintained in a password-protected file. The information was kept confidential and was not subject to individual disclosure but was included in the research report as part of the overall results. All participants who completed the questionnaires of this study received 100 Thai Baht (US $3) as compensation for their time.

### Statistical Analysis

All statistical analyses were performed using SPSS (IBM Corp). Descriptive analyses were performed to assess demographic data, telehealth service use, influencing factors of telehealth service use, and health outcomes. Significant differences in telehealth service use among various time frames after enrollment (month 1, month 2, month 3, and month 4) were tested using repeated measures ANOVA with a post hoc pairwise test based on Bonferroni correction. Multiple linear regression analysis with the entered method was performed to identify potential factors associated with telehealth service use. These factors were selected based on theoretical criteria. Age, health literacy, perceived usefulness, employment status, perception of illness, and patient-provider relationship were added as independent variables, and the use of the telehealth service was added as the dependent variable. A simple linear regression analysis was performed to determine the relationship between the rates of telehealth service use and quality of life. Additionally, logistic regression was applied to understand the relationship between the rates of telehealth service use and peritonitis.

## Results

### Participants’ Characteristics

A total of 159 patients undergoing CAPD and using the telehealth service for 4 months were included in this study. The mean age of the patients was 55.7 (SD 13.1) years, and the mean time on CAPD was 2.9 (SD 2.2) years. Of the 159 patients, 82 (51.6%) were women, 89 (56%) were married, 68 (42.8%) had completed primary school-level education, 120 (75.5%) were employed, and 131 (82.4%) were under universal coverage (Table 1).

**Table 1:** Demographic information of participants (n=159). A currency exchange rate of THB 1=US $0.02845 is applicable.

Characteristics				Values, n (%)
**Age (years)**				
	<30			5 (3.1)
	30-45			26 (16.4)
	46-60			68 (42.8)
	>60			60 (37.7)
**Gender**				
	Women			82 (51.6)
	Men			77 (48.4)
**Marital status**				
	Single			36 (22.6)
	Married			89 (56.0)
	Widowed, separated, or divorced			34 (21.4)
**Monthly household income (THB)**				
	≤15,000			56 (35.2)
	15,001-30,000			67 (42.1)
	>30,000			36 (22.7)
**Education**				
	No school attendance			8 (5.0)
	Primary school			68 (42.8)
	Secondary school and diploma			55 (34.6)
	Bachelor’s or higher degree			28 (17.6)
**Employment status**				
	Unemployed			39 (24.5)
	**Employed**			120 (75.5)
		Employee		61 (50.8)
		Self-employed business and others		59 (49.2)
**Medical payment method**				
	Universal coverage			131 (82.4)
	Social security scheme			23 (14.5)
	Private health insurance, self-payment, and others			5 (3.1)
**Comorbidity**				
	None			11 (6.9)
	**Yes**			148 (93.1)
			Hypertension	129 (87.2)
			Diabetes mellitus	75 (50.7)
			Dyslipidemia	32 (21.6)
			Cardiovascular diseases	12 (8.1)
			Systemic lupus erythematosus	4 (2.7)
			Others	23 (15.5)

### Telehealth Service Use

Table 2 shows the rates of telehealth service use over time and an analysis of telehealth use during the 4 months. The analysis of telehealth use revealed significant differences among the first 3-month intervals. The results indicated a daily decline in telehealth service use over time based on months after enrollment.

**Table 2:** Differences in telehealth service use over 4 months.

Time			Telehealth service use, mean (SD)		Mean difference		SE		*P* value		95% CI
**Month 1**			23.48 (16.28)								
	Months 1-2	N/A^a^		8.522		0.725		<.001		6.584 to 10.460	
	Months 1-3	N/A		12.396		0.887		<.001		10.026 to 14.767	
	Months 1-4	N/A		10.956		1.127		<.001		7.944 to 13.968	
**Month 2**			14.96 (13.35)								
	Months 2-3	N/A		3.874		0.508		<.001		2.517 to 5.231	
	Months 2-4	N/A		2.434		0.870		.04		0.110 to 4.758	
**Month 3**			11.09 (11.48)								
	Months 3-4	N/A		−1.440		0.838		.53		−3.680 to 0.799	
Month 4			12.53 (14.62)								

^a^N/A: not applicable.

### Factors Influencing Telehealth Service Use

Multiple linear regression analysis was performed to identify the factors influencing telehealth service use. The findings revealed that all independent variables jointly explained 27.6% of the sample variances in telehealth service use (*R*^2^=0.276; *F*_6,152_=9.654; *P*<.001). Older age (β=.221; *P*=.002), higher perceived usefulness (β=.414; *P*<.001), unemployment (β=−.155; *P*=.03), and positive illness perception (β=−.205; **P*=*.004) were associated with a significantly higher rate of telehealth service use (Table 3).

**Table 3:** Factors influencing telehealth service use.

Variables	Mean (SD)	B^a^	SE	β^b^	*t* test (152)	*P* value
Constant	—^c^	−23.591	47.422	—	−0.497	.62
Age (years)	55.7 (13.1)	0.840	0.271	.221	3.101	.002
Health literacy	3.0 (0.4)	1.429	7.976	.014	0.179	.86
Perceived usefulness	16.7 (2.2)	9.099	1.762	.414	5.165	<.001
Employment status (reference: unemployed)	—	−17.862	8.143	−.155	−2.194	.03
Perception of illness	39.2 (10.6)	−7.641	2.616	−.205	−2.921	.004
Patient-provider relationship	37.9 (4.3)	−9.542	7.949	−.094	−1.200	.23

^a^B: unstandardized beta.

^b^β: standardized beta.

^c^Not available.

### Telehealth Service Use and Health Outcomes

Regarding the relationship between the rates of telehealth service use and quality of life, the result showed that the higher the rates of telehealth service use, the better the quality of life (β=.241; *P*=.002; Table 4). Additionally, logistic regression analysis revealed that the higher the rates of telehealth service use, the lower the rates of peritonitis in patients undergoing CAPD (odds ratio 0.980, 95% CI 0.962-0.997; *P*=.03; Table 5).

**Table 4:** Simple linear regression of factors influencing the quality of life.

Variable	Mean (SD)	B^a^	SE	β^b^	*t* test (157)	*P* value
Constant	—^c^	170.108	6.542	—	27.223	<.001
Telehealth service use	194.05 (52.87)	0.257	0.082	.241	3.118	.002

^a^B: unstandardized beta.

^b^β: standardized beta.

^c^Not available.

**Table 5:** Logistic regression of factors influencing peritonitis.

Variable	B^a^	SE	*df*	*P* value	OR^b^ (95% CI)
Constant	–1.493	0.416	1	<.001	0.225 (0.099-0.508)
Telehealth service use	–0.021	0.009	1	.03	0.980 (0.962-0.997)

^a^B: unstandardized beta.

^b^OR: odds ratio.

## Discussion

### Principal Findings and Implications

In this study, the rates of telehealth service use in patients undergoing CAPD for over 4 months after enrollment in the RCT study were examined. The results showed that the rates of telehealth service use declined significantly over time. This finding is consistent with those from previous studies on patients undergoing PD and having other chronic conditions [22,27,70]. Possible explanations for this finding might be loss of interest and hidden costs [71] associated with the telehealth service (internet cost). Previous studies suggested that lower income led to lower rates of telehealth use [14,56,72]. In this study, the participants were mostly from low-income groups. In either explanation, the manual data entry burden might have impacted the continuous use of telehealth services [71,73].

Regarding the factors influencing telehealth service use, predisposing characteristics (age and perceived usefulness), enabling resources (employment status), and need factors (perception of illness) significantly influenced its use. In this study, older age was a significant influencing factor of telehealth service use. This finding agrees with those from previous studies that older patients were more likely to adhere to the use of telehealth services than younger patients [28,37,38]. Older or retired patients might have more time for self-care and management than younger patients with a tight work schedule. With regard to the older participants, simple telehealth service and basic technological devices (smartphones) should be designed to promote telehealth service use in this subgroup. Additionally, special attention should be given to younger patients with CAPD to integrate the telehealth service into their daily lives. Adherence reminders to use the telehealth service and empowerment in this group should be established to enhance the use rate of telehealth service.

This study confirmed that perceived usefulness was an explanatory factor that exerted the most significant effect on telehealth service use, which is consistent with the results reported in several previous studies [18,19,35,52,74,75]. Based on the theoretical framework, the more people value telehealth services, the more they use them. This telehealth service was developed based on users’ needs assessed from focus group interviews before the development of the application [12]. Patients with CAPD and their caregivers at the dialysis center provided useful information on which telehealth service should be developed to support their dialysis treatment. Resources on knowledge and skills related to PD and self-care, communication channels in various forms, notifications of abnormalities and treatment dues, dialysis fluid follow-up, recording, and monitoring of health- and dialysis-related information were required [12]. Finally, the service comprised several features to support the user’s daily health, which fulfilled their needs. Moreover, this telehealth service was delivered by a multidisciplinary team from the PD center, which made the participants receive care and connect digitally with the center. These factors might make the patients feel satisfied [13,76-78] and perceive the service as useful. Thus, they were more likely to use the telehealth service regularly. Therefore, the usefulness of the telehealth service should be established through a process of designing and developing it. Need assessment should be primarily done to understand the requirements of particular users. A proper architecture design that targets end users’ needs for both functionality and usability should be developed.

Employment status was another key influencing factor in telehealth service use. Unemployed participants, especially retired groups, tend to use the telehealth service more frequently than employed ones. Based on the theoretical framework [30], if resources are available, which was defined as time available in this study, they can use health services regularly. The telehealth service is adopted as daily telemonitoring that enables patients to record and transfer their personal health data to the PD center. The participants who rarely used the service stated that they were too busy with their work and responsibilities. This result is consistent with a previous study showing that time constraints and efforts were barriers to the continuous use of telehealth services [79]. Thus, the data entry burden should be considered when delivering telehealth services to this group of people. Our finding alludes to the idea that suitable telehealth service features should be explored for different user groups in terms of employment status and time availability to increase the rates of use. The use of passive monitoring, such as wearable devices, may further reduce the participants’ burden of data entry.

In this study, perception of illness was the least significant factor contributing to telehealth service use. Participants perceiving less severe illnesses were more likely to use the telehealth service. Certain findings from this study are comparable to those reported in previous studies, which showed that patients with poor health conditions, high levels of comorbidity, or low baseline health were less likely to adopt health-related technology, including telehealth [27,29,55,57,72,80]. With the increase in the severity of illness, user capacity to adhere to telehealth service use may be limited [57,80,81]. The functional ability might be reduced in participants with illnesses that limit their capability to use health technology. Conversely, participants in good condition might find it easy to use and adhere to the telehealth service, which might enhance the use of this health service. Thus, a higher perception of risks or health threats may hinder the adoption of health care technology. It is worth mentioning that not every patient with chronic illness can handle telehealth service through mobile health apps. Patients with physically active or asymptomatic phases of their illness may be more likely to use this health care technology to prevent long-term complications and ensure safety at home. A tailored and user-friendly telehealth service should be established to enhance the rate of use of the service. In addition, family members or caregivers should support the use of this technology in patients with more severe health issues to maintain and augment the effective use of the telehealth service.

Furthermore, this study evaluated the relationships between the rates of telehealth service use and health outcomes, including quality of life and peritonitis, in this population. The results showed that higher rates of telehealth service use had a positive impact on quality of life. These findings are comparable to those from previous studies on patients undergoing PD [15,16,82,83]. Increased adherence to telehealth service use could support patients in performing CAPD safely at home, maintaining compliance with dialysis prescription, monitoring any complications, providing health information, and improving their lifestyle while receiving CAPD, thereby enhancing their quality of life. Moreover, the rates of telehealth service use could also predict peritonitis development in this population. The study results revealed that the higher the rates of telehealth service use, the lower the rates of peritonitis. This finding agrees with a previous study on Thai people undergoing CAPD [82]. By offering assistance and encouragement, the telehealth service through mobile health apps can allow health care providers to monitor, assess, and address potential or real hazards that may increase the risk of infection and reaffirm the participant’s confidence in self-care. Using mobile health apps to support CAPD management at home has been identified to increase the early diagnosis of infection [84,85]. Therefore, patients who have been supported to perform CAPD safely and have been provided with educational materials through the telehealth service could effectively perform CAPD at home, and infections, especially peritonitis, could be reduced. Patients should be motivated to use and adhere to the telehealth service, which could in turn improve their health outcomes.

This study has important theoretical and practical implications. Theoretically, the factors influencing the use of telehealth services have been investigated using the Andersen behavioral model, which has contributed to expanding the theoretical scope of this model. Additionally, this study has provided valuable insights into the influential factors for patients undergoing CAPD, which are likely to be beneficial for increasing the use of telehealth services in this patient group. Unlike other studies related to telehealth service use, this study has paid attention to the actual and continued use of this service, which is important for the success of health care technologies, particularly in the context of a developing country. This research has further examined the consequences of using the telehealth service, in contrast to previous studies that were focused on identifying the determinants of use intention or actual use behavior.

Regarding the practical implications, the findings of this study can be used by telehealth service developers, health care providers, and health-related institutions in Thailand and other countries, particularly low- and middle-income countries, to increase the use of telehealth services. More attention should be paid to the predisposing characteristics (age and perceived usefulness), enabling resources (employment status), and needs (perception of illness) of end users in the future to develop telehealth services for enhancing the use of this technology in patients with CAPD. Telehealth service should be easy to use to encourage and enhance its use in older patients with CAPD, thereby augmenting access to care. This study can also serve as a basis for developing an effective telehealth service and determining the optimal level of its use to promote better user outcomes. Health care policy makers should consider integrating this service with standard care as it can support home-based therapy and enhance health outcomes.

### Limitations and Future Research

This study has certain limitations that should be acknowledged. The small sample size might have affected the study results. Additionally, this study measured the telehealth service use over a period of 4 months. Variations in health technology use may be limited. Another limitation is that the participants were recruited from a PD center in Bangkok, Thailand. This limits the generalization of the findings to patients undergoing CAPD with different characteristics and settings.

To better understand participants’ active use, future studies should explore more factors that influence telehealth service use. Furthermore, future research should compare the outcomes across countries to generalize the findings, as telehealth services vary across countries. Moreover, as this was a cross-sectional study, the results may change over time because of the changes in both user behaviors and telehealth services. Thus, longitudinal mixed methods research studies are needed to address this issue.

### Conclusions

The findings from this study indicated that the rates of telehealth service use in patients undergoing CAPD declined over time. Older age, higher perceived usefulness, unemployment, and positive illness perception were significant factors influencing the telehealth service use. Furthermore, this study observed that adherence to telehealth service use could significantly improve the quality of life and reduce the peritonitis rate in patients undergoing CAPD.

## Abbreviations

CAPD: continuous ambulatory peritoneal dialysis
PD: peritoneal dialysis
RCT: randomized controlled trial

## Data Availability

The data sets generated and analyzed during this study are available from the corresponding author upon reasonable request.
